# Electric-field modulated energy transfer in phosphorescent material- and fluorescent material-codoped polymer light-emitting diodes

**DOI:** 10.1039/d4ra00669k

**Published:** 2024-04-16

**Authors:** Ling-Chuan Meng, Yan-Bing Hou

**Affiliations:** a Key Laboratory of Luminescence and Optical Information, Ministry of Education, Institute of Optoelectronic Technology, Beijing Jiaotong University Beijing 100044 PR China lcmeng@bjtu.edu.cn

## Abstract

The excited-state energy transfer widely exists in mixed-material systems and devices. The modulation of an electric field on the energy transfer in photoluminescence has been demonstrated. However, to date, no studies on the electric-field modulation of the excited-state energy transfer in organic optoelectronic devices have been reported. Herein, we investigate the effect of an electric field on the energy transfer in the poly(*N*-vinylcarbazole) (PVK) thin films doped with iridium(iii)[bis(4,6-difluorophenyl)pyridinato-N,C^2′^]-tetrakis(1-pyrazolyl)borate (Fir6) and 5,6,11,12-tetraphenylnaphthacene (rubrene) (PVK:Fir6:rubrene) and the corresponding light-emitting diodes. Combined with the Onsager model describing electric-field enhanced exciton dissociation, we find that the electric field increases the rate of Dexter energy transfer from Fir6 to rubrene in the films and the diodes. The voltage-dependent color shift in the PVK:Fir6:rubrene light-emitting diodes can be explained by the electric-field enhanced Dexter energy transfer from Fir6 to rubrene. Our findings are important for the control of energy transfer process in organic optoelectronic devices by an electric field for desirable applications.

## Abstract 2

In this study, we find electric-field modulated energy transfer in phosphorescent material- and fluorescent material-codoped polymer that has never been reported.

## Graphical abstract

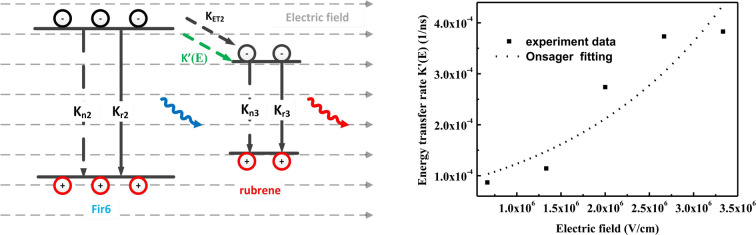

## Introduction

The excited-state energy transfer in multi-material systems, which is defined as a physical process in which the energy of a photo- or electro-excited donor is transferred to an acceptor in the ground state, widely exists in organic light-emitting didoes (OLEDs),^1–3^ organic solar cells,^4,5^ up-conversion luminescence,^6,7^ fluorescence probes,^8,9^ energy transfer catalysis,^10,11^ and photochemical synthesis.^12–15^ In mixed systems of luminescent conjugated polymers, organic dyes, and semiconducting quantum dots^13,16–20^ energy transfer has been extensively studied, where the precise control of energy transfer process is of vital importance in achieving desirable photoluminescence (PL) characteristics. Upon the application of an external electric field, energy transfer can be modulated because the electric field tunes the spatial distributions of both electrons and holes, as well as their interactions.^21^ The effect of electric-field manipulation on Förster resonant energy transfer has been observed in photo-excited polyfluorene,^13^ which is monitored by enhanced bimolecular annihilation *via* the increased overlap between the singlet emission and absorption spectra due to Stark effect. The electrically switchable nanocrystal–dye couples consisting of CdSe/CdS nanorods and 1,1′,3,3,3′,3′-hexamethylindodicarbocyanine iodide have been demonstrated at 50 K. The fluorescence of the couples is modulated by cyclic application of a bias because the CdSe/CdS nanorods exhibit a large quantum-confined Stark effect on the single-particle level, enabling the direct electrical control of the spectral resonance between the rods and the dye required for the nanoscopic Förster-type energy transfer from a single semiconductor nanorod to a dye molecule in the couples.^19^ Recently, enhanced upconverted circularly polarized luminescence based on an electric-field regulated radiative energy transfer process from NaYF_4_:Yb/Tm up-conversion nanoparticles to CsPbBr_3_ perovskite nanocrystals has been successfully implemented by blending the nanomaterials in a chiral nematic liquid crystal (SLC1717), which can serve as an electric-field controlled upconverted circularly polarized luminescence switch.^22–24^

In electroluminescence (EL), the investigations on the process of the excited-state energy transfer plays a key role in elucidating the working mechanisms of OLEDs and tuning the EL properties.^25,26^ The energy transfer between organic fluorescent and phosphorescent materials has been utilized to reduce energy losses, improve EL efficiency, and realize white light emission.^1–3^ Fluorescent OLEDs with a high efficiency have been demonstrated by using a phosphorescent sensitizer *fac*-tris(2-phenylpyridine)iridium (Ir(ppy)_3_) to excite a fluorescent dye [2-methyl-6-[2-(2,3,6,7-tetrahydro-1*H*,5*H*-benzo[*ij*]quinolizin9-yl)ethenyl]-4*H*-pyran-4-ylidene] propane-dinitrile (DCM2) that are doped in alternating thin layers of 4,4′-*N*,*N*′-dicarbazole-biphenyl (CBP), benefiting from the long-range, non-radiative Förster energy transfer process involved in the energetic coupling between the phosphorescent and fluorescent molecular species.^27^ Highly-efficient white OLEDs with a fluorescent blue emitter *N*,*N*′-di-1-naphthalenyl-*N*,*N*′-diphenyl[1,1′:4′,1′′:4′′,1′′′-quaterphenyl]-4,4′′′-diamine (4P-NPD) sandwiched between a phosphorescent orange emitter system [iridium(iii)bis(2-methyldibenzo-[*f*,*h*]quinoxaline)(acetylacetonate) (Ir(MDQ)_2_(acac)) doped α-NPD and a phosphorescent green one [Ir(ppy)_3_ doped 2,2′,2′′(1,3,5-benzenetriyl) tris-[1-phenyl-1*H*-benzimidazole] (TPBi) have been introduced. The novel concept for these devices lies in that both electrically generated singlet and triplet excitons are employed for light generation *via* Förster and Dexter energy transfer paths between the fluorescent emitters and the phosphorescent iridium complexes,^28^ which can lead to a total internal quantum efficiency of almost 100%. Besides, in the white OLEDs based on a wide-bandgap host material 1,3-bis(9-carbazolyl)benzene (mCP) doped with phosphorescent dyes iridium(iii)[bis(4,6-difluorophenyl)pyridinato-N,C^2′^]picolinate (FIrpic) for blue emission and bis(2-(9,9-diethyl-9*H*-fluoren-2-yl)-1-phenyl-1*H*-benzoimidazol-N,C^3^)iridium(acetylacetonate) ((fbi)_2_Ir(acac)) for orange emission, the careful manipulation of host–guest energy transfer for the blue dopant and direct exciton formation for the orange dopant ensures the harvest of excitons *via* two parallel channels for efficient white emission with nearly 100% internal quantum efficiency.^29^ Similar to the modulation of an electric field on energy transfer in PL, it is possible that the energy transfer in OLEDs would be affected by an applied electric field. This might lead to a change of the EL spectrum with increasing applied voltages or electric fields, namely a voltage-dependent color shift,^30^ which is known to be a big challenge for white OLEDs in practical applications. However, to date, no studies on the electric-field modulation of the excited-state energy transfer in OLEDs have been reported.

In this work, we investigate the effect of an electric field on the energy transfer in phosphorescent material- and fluorescent material-codoped polymer light emitting diodes. We prepared poly(*N*-vinylcarbazole) (PVK) thin films doped with iridium(iii)[bis(4,6-difluorophenyl)pyridinato-N,C^2′^]-tetrakis(1-pyrazolyl)borate (Fir6) and 5,6,11,12-tetraphenylnaphthacene(rubrene) (PVK:Fir6:rubrene) and fabricated the light-emitting diodes based on the PVK:Fir6:rubrene films. The impact of an electric field on the energy transfer between Fir6 and rubrene in the PL process of the PVK:Fir6:rubrene films was explored by steady-state PL spectra in electric field and electric field modulated transient PL spectra. Combined with the Onsager model describing electric field enhanced exciton dissociation, we find that the electric field increases the rate of Dexter energy transfer in the films and interpret its physical mechanism. This effect also exists in the PVK:Fir6:rubrene light-emitting devices, which explains the dependence of EL spectrum on applied voltage.

## Experimental

**Fig. 1:**
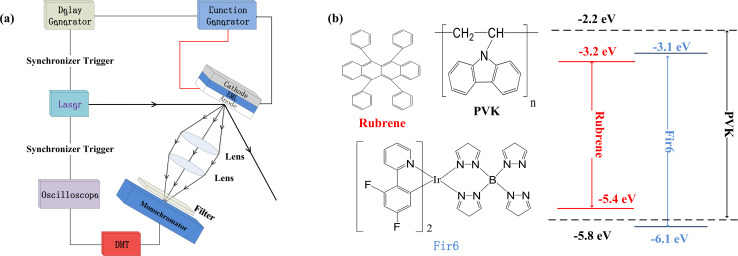
(a) Schematic diagram of the home-made setup for the measurement of the electric-field modulated transient PL spectra. PMT is a photomultiplier tube. A 355 nm pulsed helium-neon laser with a width of 5 ns and frequency of 10 Hz was used as the excitation light source. To improve the response speed of the setup so that the response time can be much less than the excited state lifetime of the sample and to achieve a sufficiently strong sampling signal, a 50 ohms sampling resistance was utilized for the oscilloscope. The parasitic capacitance of the oscilloscope is 100 pF. Therefore, the time constant of this setup is around 5 ns, which is much shorter than the phosphorescent lifetime (about 1000 ns) of Fir6 in the PVK:Fir6:rubrene thin films; (b) the molecular structures of PVK, Fir6, and rubrene, as well as their energy level alignment.

We prepared the PVK:Fir6:rubrene thin films on quartz substrates, and fabricated the OLEDs in a structure of ITO/PEDOT:PSS/PVK:Fir6:rubrene/Ca/Al. The quartz substrates and ITO-glass substrates were ultrasonically cleaned with anhydrous ethanol, acetone, and deionized water in sequence. During the film preparation, PVK, Fir6 and rubrene in different ratios were dissolved in chloroform, and then the solutions were spun onto the quartz substrates. When fabricating the OLEDs, the pre-filtered PEDOT:PSS(4083) aqueous solution was first spin coated on the ITO glass substrate in ambient conditions, followed by the deposition of the PVK:Fir6:rubrene thin film and annealing treatment. Finally, in a high vacuum environment of about 10^−7^ Torr a 20 nm calcium film and a 100 nm aluminum film were deposited by thermal evaporation method, serving as cathodes. The effective emission area of the device is 9 mm^2^, which is defined by a shadow mask. The absorption spectra were measured by Shimadzu 3101PCUV-Vis-NR spectrophotometer. The PL spectra were recorded using SpexFluorolog-3 fluorescence spectrophotometer. In order to reveal the effect of an electric field on energy transfer, the measurements of electric-field modulated steady-state PL spectra and transient PL spectra were carried out. Fig. 1(a) shows schematic diagram of the home-made setup for the electric-field modulated transient PL spectra.

## Results and discussion

**Fig. 2:**
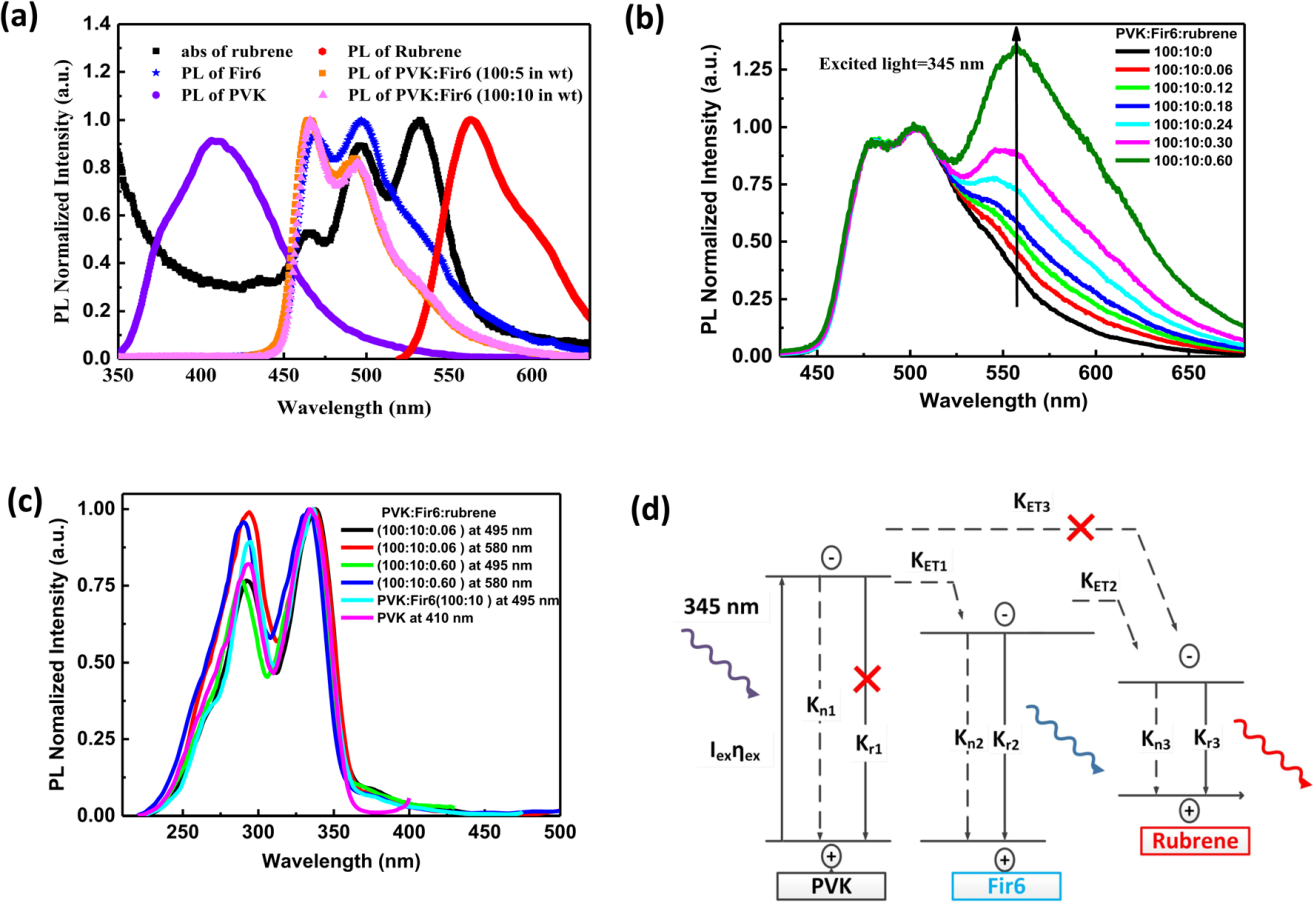
(a) PL spectra of PVK, Fir6, rubrene, and the PVK:Fir6 thin films at different weight (wt) ratios and absorption of rubrene. Abs is the abbreviation of absorption; (b) PL spectra of the PVK:Fir6:rubrene (100 : 10 : *X* in wt) thin films. *X* stands for the weight content of rubrene; (c) PL excitation spectra of the PVK, PVK:Fir6, and PVK:Fir6:rubrene thin films. The monitored wavelengths are 410 nm for PVK, 495 nm for Fir6, and 580 nm for rubrene, respectively; (d) exciton dynamics of the PVK:Fir6:rubrene thin films under photoexcitation. *I*_ex_ represents the excitation laser intensity. *η*_ex_ is the absorption coefficient of PVK. *K*_n1_, *K*_n2_, and *K*_n3_ represent the non-radiative recombination rates of PVK, Fir6, and rubrene, and *K*_r1_, *K*_r2_, and *K*_r3_ stand for the radiative recombination rates of PVK, Fir6, and rubrene, respectively. *K*_ET1_ is the energy transfer rate from PVK to Fir6, *K*_ET2_ is the energy transfer rate from Fir6 to rubrene, and *K*_ET3_ is the energy transfer rate from PVK to rubrene. The symbol “×” indicates that the process can be ignored or is really weak.

Fig. 1(b) illustrates the molecular structures and energy level diagrams of PVK,^31,32^ Fir6,^33^ and rubrene.^34^ The commonly used blue phosphorescent Fir6 as the energy donor and highly efficient fluorescent rubrene as the energy acceptor were co-doped in the large bandgap polymer host PVK, forming a single-layer organic light-emitting system where energy transfer was involved. The bandgaps of the host PVK, the phosphorescent dopant Fir6 and the fluorescent dopant rubrene are 3.6, 3.0, and 2.2 eV, respectively, which sequentially decreases, benefiting to the occurrence of energy transfer in this mixed-material system. Specifically, the Lowest Unoccupied Molecular Orbital (LUMO) level of Fir6 (−3.1 eV) is higher than that of rubrene (−3.2 eV) and the Highest Occupied Molecular Orbital (HOMO) level of Fir6 (−6.1 eV) is lower than that of rubrene (−5.4 eV), which can ensure the energy transfer from Fir6 to rubrene. According to the theory of energy transfer, the overlap area between the emission spectrum of an energy donor and the absorption spectrum of an energy acceptor is an important factor for energy transfer rate. Fig. 2(a) displays the PL spectra of PVK, Fir6, and rubrene and the absorption spectrum of rubrene. The emission peak of PVK is located at 410 nm, and those of Fir6 are at 465 and 495 nm. The absorption peaks of rubrene are at 465, 495, and 540 nm, respectively. The obvious larger overlap between the PL spectrum of Fir6 and the absorption spectrum of rubrene in the wavelength range from 450 to 550 nm indicate strong energy transfer from Fir6 to rubrene. However, the overlap between the emission spectrum of PVK and the absorption spectrum of rubrene is much smaller, which suggests much weaker energy transfer from PVK to rubrene comparing to the energy transfer from Fir6 to rubrene.

On the other hand, the energy transfer from PVK to Fir6 was investigated by comparing the PL spectrum of the PVK film and the ones of the PVK:Fir6 thin films with different Fir6 concentrations. The PL spectra of the PVK:Fir6 (100 : 5 in wt) and PVK:Fir6 (100 : 10 in wt) films shown in Fig. 2(a) exhibit the same emission peaks at 465 and 495 nm, which are consistent with the intrinsic ones of Fir6, while no characteristic emission of PVK is observed. This suggests that the energy of the excited state of PVK can transfer to Fir6 so effectively that PVK itself does not emit any light when the ratio of PVK to Fir6 is in the range from 100 : 5 to 100 : 10 in wt. Therefore, the ratio of 100 : 10 in wt is chosen in the following study to ensure that the effective energy transfer from PVK to Fir6 works.

In order to study the energy transfer from the energy donor Fir6 to the energy acceptor rubrene in the PVK:Fir6:rubrene thin films, the rubrene concentration should be low enough to avoid the energy transfer from PVK to rubrene. Meanwhile, it should ensure that the PL intensity of rubrene is comparable to that of Fir6 so that it is easy to observe the spectral change caused by the change in energy transfer. The steady-state PL spectra of the PVK:Fir6:rubrene (100 : 10 : *X* in wt) (*X* = 0.06, 0.12, 0.18, 0.24, 0.30, 0.60, 0.90, 1.20, 1.50, 1.80, 2.10, 2.40) thin films with different rubrene concentrations were measured, as shown in Fig. 2(b). All the films show three similar emission peaks locating at about 465, 495, and 580 nm. The former two peaks correspond to the intrinsic emission of Fir6, and the latter originates from rubrene. This reveals efficient energy transfer between Fir6 and rubrene in the PVK:Fir6:rubrene. A little red shift in PL spectrum peak at 580 nm which is the emission of rubrene can be found as the doping concentration of rubrene increased. That is ascribed to solid state solvent effect.^35^ Since the energy transfer from PVK to rubrene is much weaker, the excited-state energy of rubrene in the PVK:Fir6:rubrene thin film mainly stems from the energy transferred from Fir6. This conclusion is further verified by comparing the excitation spectra of the PVK, PVK:Fir6, and PVK:Fir6:rubrene thin films as shown in Fig. 2(c). The monitored wavelengths are 410 nm for PVK, 495 nm for Fir6, and 580 nm for rubrene, respectively. It can be seen that the PL excitation spectra of the PVK:Fir6 and PVK:Fir6:rubrene films are almost the same as the one of PVK, indicating that the excited-state energy of Fir6 and rubrene both come from the excited PVK in the mixed-material system. Considering the presence of effective energy transfer from PVK to Fir6 when the ratio of PVK:Fir6 is 100 : 10 in wt as discussed above, the much weaker energy transfer from PVK to rubrene than that from Fir6 to rubrene for the much smaller spectral overlap area between emission of PVK and absorption of rubrene than that between Fir6 and rubrene as discussed above, as well as the much lower rubrene doping concentration in PVK (0.3%) than that of Fir6 (10%), it's reasonable to know almost all energy of excited PVK was transferred to Fir6 instead of rubrene. Upon the discussions above, it can be concluded that the majority of the excited-state energy of rubrene in the PVK:Fir6:rubrene film results from the energy transfer from Fir6 to rubrene. In addition, the emission intensity ratio of rubrene to Fir6 increases with the rubrene concentration, which suggests that the energy transfer from Fir6 to rubrene is enhanced with increasing the rubrene molecules. According to the above results, we depict the exciton dynamics of the PVK:Fir6:rubrene thin films under photoexcitation in Fig. 2(d). When the film is illuminated by an excitation light of 345 nm, the PVK molecules absorb most of the energy of excitation light and are in excited states. Then the excited states of PVK molecules decay by efficiently transferring the energy to the Fir6 molecules at a rate of *K*_ET1_, rather than decay radiatively. The excited Fir6 molecules could decay in three ways. Partial excitons of Fir6 decay radiatively to emit blue light whose emission peaks are 465 and 495 nm and partial relax to the ground states nonradiatively, whereas the energy of the rest excitons transfers to rubrene at a rate of *K*_ET2_. Finally, some of the excited rubrene molecules decay to the ground states radiatively to emit yellow light peaking at 580 nm and others relax nonradiatively. It cannot be ruled out that a small portion of energy in the excited PVK molecules would excite the rubrene molecules through energy transfer. However, as discussed above, this part of energy can be neglected compared to the energy transferred from PVK to Fir6. With increasing the rubrene concentration, the energy transfer from Fir6 to rubrene enhances, and more rubrene molecules are excited, resulting in an increase in the emission intensity of rubrene.^36^ Moreover, as shown in Fig. 1(b), the phosphorescence intensity of Fir6 is comparable to the fluorescence intensity of rubrene in the PVK:Fir6:rubrene (100 : 10 : 0.30 in wt) thin film, which makes it suitable for the study of the energy transfer mechanism between Fir6 and rubrene in an electric field.

**Fig. 3:**
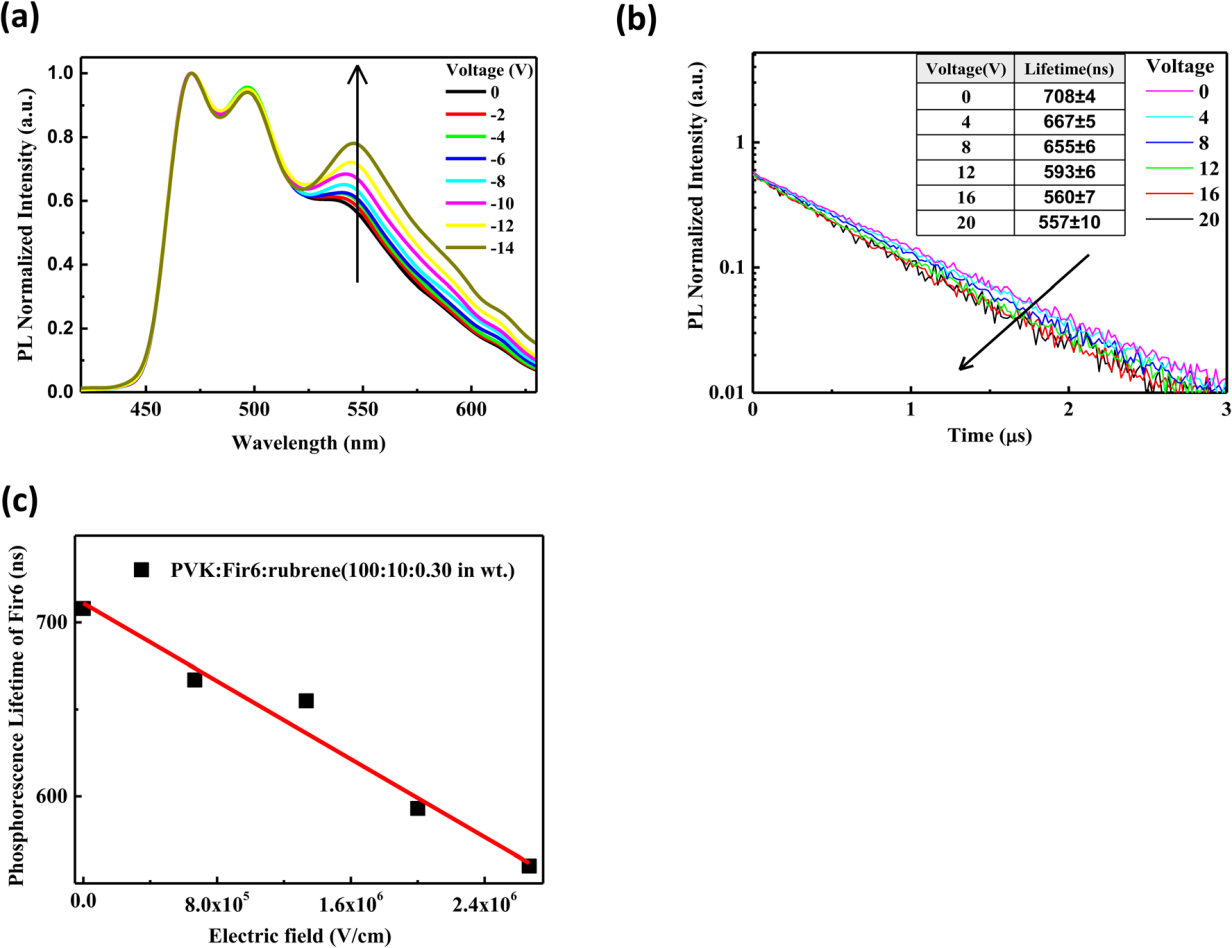
(a) Steady-state PL spectra of the PVK:Fir6:rubrene (100 : 10 : 0.30 in wt) thin film in reverse electric fields with a structure of ITO/PVK:Fir6:rubrene (100 : 10 : 0.30 in wt)/Al. The ITO electrode is negatively biased; (b) transient PL spectra of the PVK:Fir6:rubrene (100 : 10 : 0.30 in wt) thin film in reverse electric fields with a structure of ITO/PVK:Fir6:rubrene (100 : 10 : 0.30 in wt)/Al. The ITO electrode is negatively biased; (c) variation of the phosphorescence lifetime of Fir6 with the electric field in the PVK:Fir6:rubrene (100 : 10 : 0.30 in wt) thin film.

For a phosphorescent- and fluorescent-material-codoped polymer thin film, its electroluminescence is a result of the combined effect of both energy transfer and direct charge trapping. To avoid the disturbance of charge trapping effect during investigating the effect of an electric field on the energy transfer between Fir6 and rubrene in the LEDs based on the PVK:Fir6:rubrene system, we first explored the influence of an electric field on the energy transfer behavior of the PL process in the PVK:Fir6:rubrene (100 : 10 : 0.30 in wt) thin film. In the case of optical excitation without carrier injection, charge trapping effect does not contribute to the PL of the PVK:Fir6:rubrene film. The excited-state energy of Fir6 and rubrene in the PVK:Fir6:rubrene thin films mainly comes from the energy transfer among PVK, Fir6, and rubrene based on the exciton dynamics without an electric field (Fig. 2(d)), thus, it is reasonable to study the impact of an electric field on the energy transfer from Fir6 to rubrene by exploring the photoluminescence spectra of the PVK:Fir6:rubrene films under bias. Fig. 3(a) shows the steady-state photoluminescence spectra of the PVK:Fir6:rubrene (100 : 10 : 0.30 in wt%) film in electric fields with a structure of ITO/PVK:Fir6:rubrene (100 : 10 : 0.30 in wt)/Al. To hinder the carrier injection from the electrodes, reverse voltages were applied to the device with ITO negatively biased. It is obvious that the relative luminescence intensity of the rubrene at 580 nm of the PVK:Fir6:rubrene film increases with increasing the applied voltage. And no red shift were found in PL spectra of the PVK:Fir6:rubrene (100 : 10 : 0.30 in wt%) film with increasing voltage. That indicates electric field's influence on exciton zone of PVK:Fir6:rubrene film under photo-excitation is too weak to be ignored. Because the excited-state energy of rubrene is mainly transferred from Fir6, it is feasible to conclude that the increment in the relative luminescence intensity of rubrene with an increasing electric field is ascribed to the enhanced energy transfer from Fir6 to rubrene induced by the electric field.

The energy transfer from Fir6 to rubrene in the PVK:Fir6:rubrene thin film is dominated by its energy transfer rate. As an important energy relaxation path for the excitons of Fir6, the energy transfer from Fir6 to rubrene has a strong impact on the phosphorescence lifetime of Fir6. Therefore, the phosphorescence lifetime of Fir6 in the film would vary with the energy transfer rate from Fir6 to rubrene. We can disclose the effect of an electric field on the energy transfer rate from Fir6 to rubrene by analyzing the change of the phosphorescence lifetime of Fir6 with the electric field. The transient photoluminescence decay curves of the PVK:Fir6:rubrene (100 : 10 : 0.30 in wt) thin film in reverse electric fields with a structure of ITO/PVK:Fir6:rubrene (100 : 10 : 0.30 in wt)/Al were recorded by the home-made setup in Fig. 1(a). The photoluminescence decay of Fir6 was monitored here. The phosphorescence lifetimes of Fir6 in the film in different electric fields were obtained by fitting the transient PL spectra with a single exponential decay model and are listed in Fig. 3(b). Fig. 3(c) presents the variation of the phosphorescence lifetime of Fir6 in the PVK:Fir6:rubrene (100 : 10 : 0.30 in wt) film with the electric field intensity. It is found that the phosphorescence lifetime of Fir6 obviously decreases as the electric field increases. This indicates that the electric field can increase the deexcitation rate of the excited states of Fir6. According to the exciton dynamics shown in Fig. 2(d), there exist three different decay pathways for excited Fir6: the radiative recombination of Fir6 at a rate of *K*_r2_, the non-radiative recombination of Fir6 at a rate of *K*_n2_, and the energy transfer from Fir6 to rubrene at a rate of *K*_ET2_. The radiative recombination rate *K*_r2_ and non-radiative recombination rate *K*_n2_ are the intrinsic properties of luminescent materials and independent of external influences.^37^ Upon the discussions above, it's reasonable to know an increased energy transfer from Fir6 to rubrene should be mainly responsible for the decreased phosphorescence lifetime of Fir6 in PVK:Fir6:rubrene thin film with applied electric field. As the electric field intensity increases, the energy transfer rate from Fir6 to rubrene increases, enabling more exciton energy of Fir6 to be transferred to rubrene. As a result, the number of the Fir6 excitons decreases, resulting in a decrease in the phosphorescent lifetime of Fir6. Meanwhile, as more rubrene molecules are excited in the PVK:Fir6:rubrene (100 : 10 : 0.30 in wt) film, the PL intensity of rubrene becomes stronger, consistent with the results of the steady-state PL spectra for the film in reverse electric fields.

**Fig. 4:**
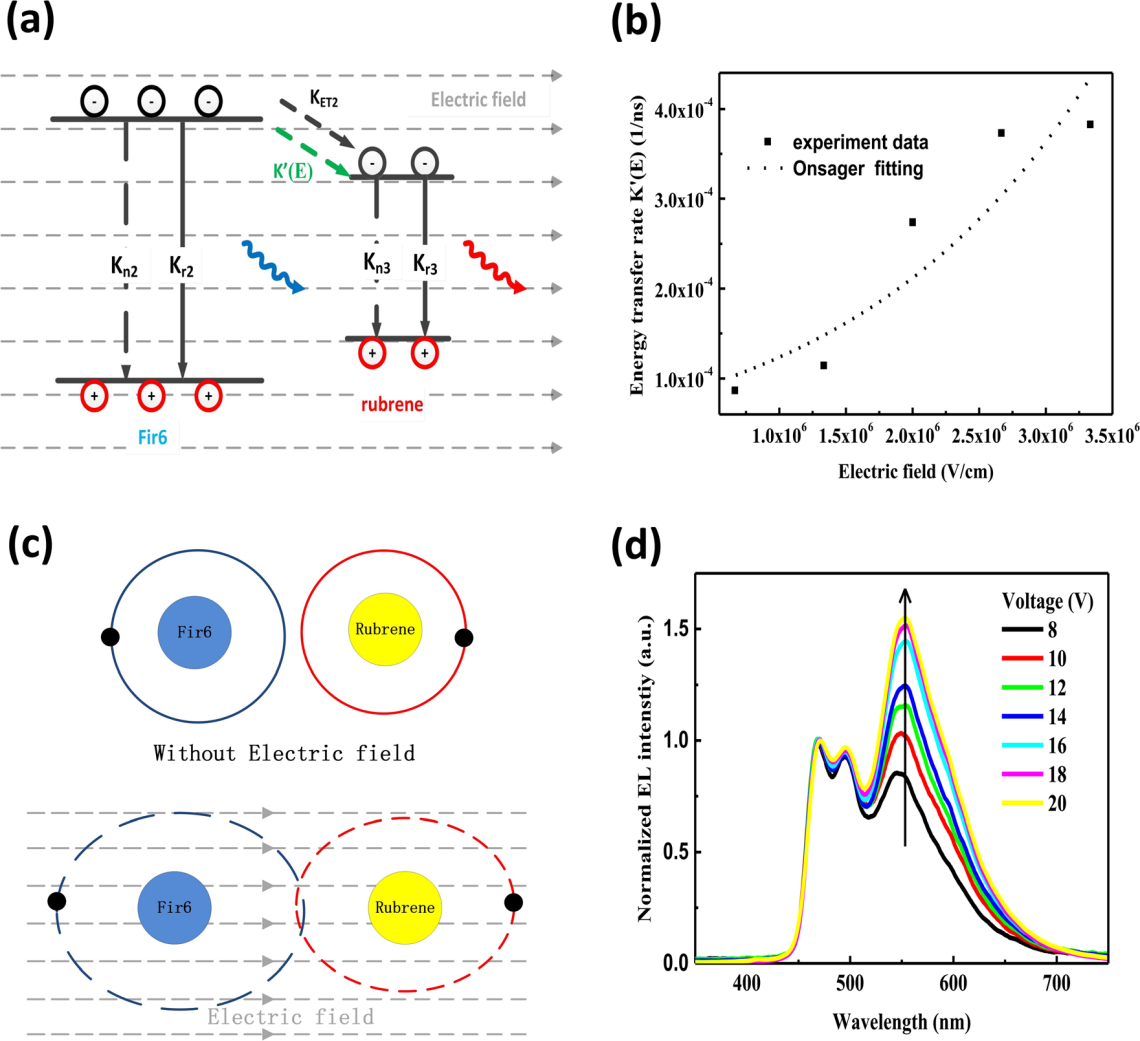
(a) Exciton dynamics of Fir6 and rubrene in the PVK:Fir6:rubrene (100 : 10 : 0.30 in wt) thin film under photoexcitation in an electric field. *K*′(*E*) is the energy transfer rate from Fir6 to rubrene that is induced by the electric field; (b) electric-field enhanced energy transfer rate from Fir6 to rubrene in the PVK:Fir6:rubrene (100 : 10 : 0.30 in wt) thin film as a function of an electric field; (c) schematic of electron cloud overlap between the Fir6 and the rubrene molecules in an electric field; (d) EL spectra of the OLED in a structure of ITO/PEDOT:PSS/PVK:Fir6:rubrene (100 : 10 : 0.30 in wt)/Ca/Al at different applied voltages.

In the following section, we conduct a detailed analysis to verify the above hypothesis that an electric field can enhance the energy transfer from Fir6 to rubrene in the PVK:Fir6:rubrene (100 : 10 : 0.30 in wt) thin film. The exciton dynamics of Fir6 and rubrene in the PVK:Fir6:rubrene (100 : 10 : 0.30 in wt) thin film under optical excitation in an electric field is shown in Fig. 4(a). When the PVK:Fir6:rubrene (100 : 10 : 0.30 in wt) film is excited by 355 nm light, but no external electric field is applied, partial excited-state energy of the Fir6 emit light *via* radiative recombination with a lifetime of *τ*, and partial is transferred to rubrene at a rate of *K*_ET2_ to excite the rubrene molecules, which then emit photons through radiative recombination. Upon the application of an electric field, the phosphorescence lifetime of Fir6 decreases from *τ* to *τ*′. It is apparent that the reduction in the lifetime is attributed to the enhanced energy transfer rate from Fir6 to rubrene, which equivalently introduces an additional energy transfer pathway at a rate of *K*′(*E*) that is modulated by the electric field. Therefore, the electric-field enhanced energy transfer rate from Fir6 to rubrene is expressed by 
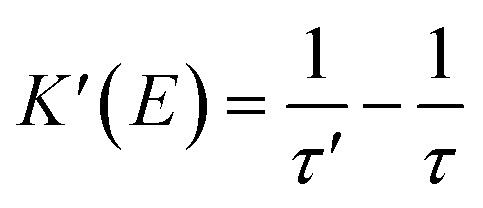
.^37–39^ From this equation and the phosphorescence lifetimes of Fir6 in Fig. 3(c), the electric-field enhanced energy transfer rates from Fir6 to rubrene in the PVK:Fir6:rubrene film are obtained, and Fig. 4(b) displays the rates as a function of the electric field. It is seen that the energy transfer rate from Fir6 to rubrene in the PVK:Fir6:rubrene film increases as the electric field increases. Through this process, more exciton energy of Fir6 is converted to the exciton energy of rubrene under the influence of the electric field, which increases the excited states of rubrene and leads to an enhancement in the PL intensity of rubrene.

According to the classical energy transfer theory, there are two fundamentally different types of energy transfer involved in organic electronics.^40^ One is Förster energy transfer and another is Dexter energy transfer. Förster energy transfer is realized between the electronic excited state of an energy donor to an energy acceptor by nonradiative dipole–dipole coupling, which greatly depends on the interactions between the transition dipole moments of the excited donor and the unexcited acceptor.^41^ The Förster energy transfer rate is determined by three factors: the distance between the donor and the acceptor, the overlap between the emission spectrum of the donor and the absorption spectrum of the acceptor, and the relative orientation of the donor dipole moment and the acceptor dipole moment. It has been reported that the effect of an external electric field on transition dipole moments is less than 0.5%.^42^ On the other hand, the distance between a donor and an acceptor is dominated by their doping concentrations and the internal structure of their host material. Therefore, an external electric field cannot significantly influence the Förster energy transfer from Fir6 to rubrene in the PVK:Fir6:rubrene (100 : 10 : 0.30 in wt) thin film. It is necessary to consider the possibility of the impact of an electric field on Dexter energy transfer, which is achieved *via* the electron cloud overlap between an energy donor and an energy acceptor.^43,44^ Dexter energy transfer is related to the wavefunction overlap between the donor and the acceptor, their distance, and the energy barrier between the excited states of the donor and the acceptor.^45^ An electric field can directly influence the electron cloud overlap and the energy barrier between the donor and the acceptor. Herein, the physical mechanism of the electric-field enhanced energy transfer from Fir6 to rubrene can be explained by the theory of electric-field induced exciton ionization,^46,47^ which is responsible for the luminescence quenching of organic films in an electric field.^48^ The average distance between the Fir6 and rubrene molecules in the PVK:Fir6:rubrene (100 : 10 : 0.30 in wt) film is estimated to be about 5 Å, which is within the range of the effective distance (5–10 Å) for Dexter energy transfer.^49^ In an external electric field, the distance between the positive center and the negative center in the Fir6 donor and the rubrene acceptor molecules increases so that the overlap between their electron clouds becomes larger, as shown in Fig. 4(c). Furthermore, the electric field can reduce the energy barrier between Fir6 and rubrene,^50^ resulting in the probability of energy transfer. Meanwhile, the electric field can promote the dissociation of the excitons in the Fir6 molecules into electrons and holes, which are involved in Dexter exchange transfer from Fir6 to rubrene *via* the electron cloud overlap. Consequently, it is highly possible that an electric field enhances the Dexter energy transfer from Fir6 to rubrene in the PVK:Fir6:rubrene thin film. The rate constant of the electric-field induced exciton dissociation is generally described by the Onsager model^51,52^ with an equation of *K*_Q_(*E*) = *K*_Q_(0)exp(*eδE*/*kT*), where *K*_Q_(0) is quenching rate of excitons without an electric field, *e* is the elementary electron charge, *k* is the Boltzmann constant, *δ* is the average distance of charge carrier hopping, *E* is the electric field strength, and *T* is the absolute temperature. By fitting the curve of the electric-field enhanced energy transfer rate as function of electric field in the PVK:Fir6:rubrene (100 : 10 : 0.30 in wt) film in Fig. 4(b) with this equation, it is found that the dependence of the electric-field energy transfer rate *K*′(*E*) from Fir6 to rubrene on the electric field strength is consistent with the one of the dissociation rate of the Fir6 excitons on the electric field, indicating that it is reasonable to ascribe the enhanced Dexter energy transfer from Fir6 to rubrene with the electric field to the electric-field induced exciton dissociation of Fir6.

Lastly, we investigate the influence of an electric field on the energy transfer in OLEDs based on the PVK:Fir6:rubrene system. To avoid influence of recombination zone (RZ) movement effect which is a big issue in multilayer OLEDs to the study,^53,54^ the simple device structure as anode/PEDOT:PSS/EML/cathode was employed for observation of EL spectrum. It's reasonable to know the excitons form at the interface between EML/cathode for two higher order mobility of hole than that of electron in organic device. For no other organic material was introduced to device as functional layer such as electron transporting layer (ETL), hole transporting layer (HTL), hole blocking layer (HBL) and so on, it's reasonable to rule out the possibility in principle that the EL spectrum and specifically the relative emission intensity of rubrene to Fir6 were intensively influenced by RZ movement effect in the device. The electroluminescence spectra of the ITO/PEDOT:PSS/PVK:Fir6:rubrene (100 : 10 : 0.30 in wt)/Ca/Al device at different applied voltages are shown in Fig. 4(d). Similar to the results of the photoluminescence of the PVK:Fir6:rubrene (100 : 10 : 0.30 in wt) thin film, even under electrical excitation with charge injection only the characteristic phosphorescence of Fir6 and fluorescence of rubrene are observed, while no emission of PVK is found. When a voltage is applied to the device, charge carriers are injected from the electrodes into the PVK:Fir6:rubrene emitting layer to form excitons on the PVK molecules. The excited-state energy of PVK is transferred to Fir6, and then to rubrene by sequential energy transfer processes (PVK→ Fir6 → rubrene). At the same time, some injected charge carriers might be directly trapped at the Fir6 and rubrene molecules, forming excitons by attracting the opposite carriers. The excited Fir6 and rubrene emit phosphorescence and fluorescence by radiative recombination, respectively. Besides, the relative emission intensity of rubrene to Fir6 is found to increase with increasing the applied voltage. This suggests there exists the phenomenon of the voltage-dependent color shift in this OLED, which can be explained by the electric-field enhanced Dexter energy transfer discussed above. With increasing the applied voltage, the rate of the Dexter energy transfer from Fir6 to rubrene increases accordingly. More rubrene molecules are excited, enhancing its fluorescence. On the other hand, the effect of charge carrier trapping at the rubrene molecules^31,55,56^ cannot be ruled out, which could influence the emission intensity of rubrene to some extent as the applied voltage increases and be responsible for the difference between PL spectrum and EL spectrum. Besides, a tiny red shift of EL peaks around 465 nm, 495 nm and 580 nm with increasing voltage could be ascribed to RZ movement towards cathode and an increment of shoulder peaks around 465 nm could be resulted from micro-cavity effect.^53,54^

## Conclusion

We have explored the effect of an electric field on the energy transfer from Fir6 to rubrene in OLEDs based on the PVK:Fir6:rubrene thin films. To avoid the disturbance of charge injection at electrodes, instead, the effect of an electric field on the energy transfer between Fir6 and rubrene in the PL process of the PVK:Fir6:rubrene film has been investigated by steady-state PL spectra under the presence of electric field and electric-field modulated transient PL spectra. The relative PL intensity of rubrene to Fir6 increases with increasing the electric field, while the phosphorescence lifetime of Fir6 decreases. These results suggest that the electric field leads to an enhancement in the energy transfer from Fir6 to rubrene in the PVK:Fir6:rubrene film. By fitting the curve of the electric-field enhanced energy transfer rate *versus* the electric field for the PVK:Fir6:rubrene film with the equation of the Onsager model that describes electric-field enhanced exciton dissociation, it is deduced that the electric field increases the rate of Dexter energy transfer in the film. This conclusion also applies to the case of electrical excitation. The voltage-dependent color shift in the PVK:Fir6:rubrene light-emitting device can be explained by the electric-field enhanced Dexter energy transfer from Fir6 to rubrene. Our findings are important for the control of energy transfer process in organic optoelectronic devices by an electric field for desirable applications.

## Author contributions

Lingchuan Meng: conceptualization, investigation, formal analysis, data curation, funding acquisition, writing – original draft. Yanbing Hou: methodology, writing – review and editing.

## Conflicts of interest

There are no conflicts to declare.

## Supplementary Material
